# Simulating the elimination of sleeping sickness with an agent-based model

**DOI:** 10.1051/parasite/2016066

**Published:** 2016-12-23

**Authors:** Pascal Grébaut, Killian Girardin, Valentine Fédérico, François Bousquet

**Affiliations:** 1 UMR177 IRD/CIRAD INTERTRYP, TA A17 G, Campus International de Baillarguet 34398 Montpellier Cedex 5 France; 2 Faculté des Sciences, Université Montpellier 2 Place Eugène Bataillon 34095 Montpellier Cedex 5 France; 3 École Normale Supérieure 15 Parvis René Descartes 69007 Lyon France; 4 UR GREEN, CIRAD, Campus International de Baillarguet 34398 Montpellier Cedex 5 France

**Keywords:** Trypanosomiasis, Tsetse flies, Agent-based model, Simulations, Transmission, Control, Elimination, Bipindi, Cameroon

## Abstract

Although Human African Trypanosomiasis is largely considered to be in the process of extinction today, the persistence of human and animal reservoirs, as well as the vector, necessitates a laborious elimination process. In this context, modeling could be an effective tool to evaluate the ability of different public health interventions to control the disease. Using the Cormas^®^ system, we developed HATSim, an agent-based model capable of simulating the possible endemic evolutions of sleeping sickness and the ability of National Control Programs to eliminate the disease. This model takes into account the analysis of epidemiological, entomological, and ecological data from field studies conducted during the last decade, making it possible to predict the evolution of the disease within this area over a 5-year span. In this article, we first present HATSim according to the Overview, Design concepts, and Details (ODD) protocol that is classically used to describe agent-based models, then, in a second part, we present predictive results concerning the evolution of Human African Trypanosomiasis in the village of Lambi (Cameroon), in order to illustrate the interest of such a tool. Our results are consistent with what was observed in the field by the Cameroonian National Control Program (CNCP). Our simulations also revealed that regular screening can be sufficient, although vector control applied to all areas with human activities could be significantly more efficient. Our results indicate that the current model can already help decision-makers in planning the elimination of the disease in foci.

## Abstract (fr)

Bien que la trypanosomiase humaine africaine soit largement considérée comme étant en voie d’extinction aujourd’hui, la persistance de réservoirs humains et animaux (ainsi que vectoriel) va compliquer le processus d’élimination de la maladie. Dans ce contexte, la modélisation peut s’avérer être un outil efficace pour évaluer la capacité des interventions de santé publique à contrôler la maladie. À l’aide du système Cormas^®^, nous avons développé HATSim, un modèle multi-agents capable d’évaluer les évolutions endémiques ainsi que la capacité des programmes nationaux de lutte à éliminer la maladie. Ce modèle prend en compte l’analyse des données épidémiologiques, entomologiques et écologiques des études de terrain menées au cours de la dernière décennie, ce qui permet de prédire l’évolution de la maladie dans cette région sur une période de 5 ans. Dans cet article, nous présentons d’abord HATSim selon les règles du protocole ODD, classiquement utilisé pour décrire les modèles en système multi-agents ; puis dans une deuxième partie, nous présentons les résultats des analyses prédictives portant sur l’évolution de la maladie du sommeil dans le village de Lambi au Cameroun, pour illustrer l’intérêt d’un tel outil. Nos résultats correspondent à ce qui a été observé par le Programme National de Lutte camerounais. Nos simulations ont également révélé que le dépistage régulier peut être suffisant, bien que la lutte antivectorielle, appliquée à tous les sites d’activités humaines, puisse être beaucoup plus efficace. Nos résultats montrent aussi que le modèle actuel peut déjà aider les décideurs dans la planification de l’élimination de la maladie dans les foyers.

## Introduction

Sleeping sickness, also known as Human African Trypanosomiasis (HAT), is a parasitic disease involving the transmission of trypanosomes by the tsetse vector *Glossina*, which only feeds on blood. Transmission of HAT generally occurs when the tsetse fly takes a blood meal from an infected mammal with *Trypanosoma brucei gambiense* (chronic form) or *Trypanosoma brucei rhodesiense* (virulent form). Nevertheless, the fly has to be competent to allow the installation and multiplication of non-infective forms of the parasite in its midgut, which is followed, after around 20 days, by migration to the salivary glands, where the trypanosomes acquire their infective capacity and can be transmitted by the injection of saliva during the bite. This ability lasts for the whole life of the tsetse fly, during which it can transmit the parasite at each blood meal. In humans, after the bite of an infective tsetse fly, the trypanosomes first multiply in blood or draining lymph nodes. At this first stage, there are no specific clinical signs and the host is considered as a reservoir. It can last from a few months to several years, until the parasites cross the blood-brain barrier and invade the central nervous system. At this second stage, neurological signs and symptoms are characteristic, but differ depending on the individual, and the course is fatal if the disease is left untreated.

Today, the disease is considered to be in the process of extinction; according to the World Health Organization, its elimination has been targeted for 2030 [29]. Nevertheless, the battle against this disease, which has prevailed for centuries in Africa, is far from over. Indeed, the maintenance of reservoirs in both humans and animals, as well as maintenance of the vector, demands a laborious elimination process [3, 12]. The tools for controlling the disease have been available for several decades, including mass screening, treatment, and vector control, although they are still being improved. This raises the question as to what the current disease status is, and why it has been difficult for National Health Programs to reach the objective of elimination. In this context, modeling could be an appropriate tool to evaluate the strength of these interventions in controlling the disease.

Trypanosomiasis was initially modeled using compartmental mathematical models with differential equations [7, 8, 16, 26]. These models revealed the possibility of self-extinction of HAT in low-prevalence foci, while also promoting the use of vector control in high-prevalence foci. However, they have also oversimplified the heterogeneity of the HAT foci and have induced computational difficulties due to the large number of foci.

In 2003, a website aimed at tsetse control (http://www.tsetse.org) was created, which proposed an interactive program (Tsetse Muse) to define vector control strategies against animal trypanosomosis. In 2004, Müller et al. [23] initiated the use of an individual-based model or agent-based model (ABM) to simulate the life cycle and behavior of each computer-generated entity in a multi-agent system. Davis et al. (2011) elaborated a constructed mechanistic model for the basic reproduction number, R0, of *Trypanosoma brucei gambiense* and *Trypanosoma brucei rhodesiense*, demonstrating the importance of the proportion of blood meals taken from humans [4]. In 2011, the website tsetse.org suggested another model for the control of tsetse flies and HAT, with an implementation tool to define the vector control device. One year later, Hargrove et al. (2012) used a Next-Generation Matrix (NGM) model to evaluate the impact on animal trypanosomosis, involving treatment with trypanocides or insecticides on cattle in Uganda [14]. More recently, Funk et al. (2013) succeeded (also with an NGM) in evaluating the importance of a wild animal reservoir in the maintenance of sleeping sickness in a Cameroonian focus [6]. Using a two-host ABM, Alderton et al. (2013) incorporated interacting agents in an abstract spatial map that included two simple daily tasks for farmer and non-farmer agents, in an attempt to predict the evolution of the *T. b. rhodesiense* and *T. b. gambiense* disease variants in northwest Uganda and southern Sudan [1].

The model we present herein is based on the methodology used by Müller et al. [23], and its development was based on the main HAT focus in Cameroon in the 2000s, the Bipindi focus. This model is different from Müller’s work by its incorporation of a spatial map corresponding to the occupation of space within this area. HATSim was developed using the Cormas^®^ system [2] that relies on an agent-based model and that was written in the Smalltalk language, using VisualWorks software^®^. This is a powerful simulation tool for improving how we understand the complex interactions between natural and social dynamics. The Overview, Design concepts, and Details (ODD) protocol that was proposed by Grimm et al. in 2006 [13] allowed us to use a standardized method to describe HATSim.

## Material and methods

### Epidemiological context and study area

**Figure 1.**
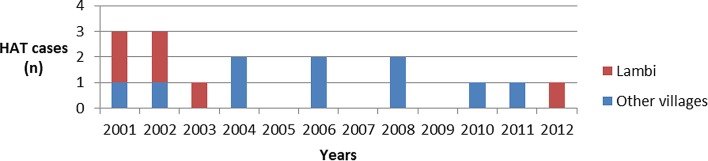
HAT cases diagnosed between 2001 and 2012 in the focus of Bipindi.

An active sleeping sickness focus in Bipindi was evident by the end of the 1990s, when 42 HAT cases were identified in four villages of southern Cameroon [10]. The prevalence reached 3.5% in two villages, Lambi and Bidjouka, which were considered to be the epicenter of the focus. At that time, 80% of the diagnosed cases were in the first phase, indicating that the disease was still expanding [10]. Regular medical surveys were conducted by the National Control Program (NCP) from 2000 to 2013, and scientific studies performed to determine the transmission by tsetse flies [5, 11, 27] suggested the circulation of *T. b. gambiense* in wild mammals [15, 24]. Nine cases in the second phase of the disease were detected from 2004 to 2012, which were all passively diagnosed (Ebo’o E.V., personal communication). Only one of these cases was diagnosed in Lambi in 2012, whereas the eight other cases were identified in other villages (Fig. 1).

The Bipindi area (Ocean Division, South Province) lies 75 km from the Atlantic coast (3N06, 10E30). The climate is equatorial of the Guinean type, with four seasons: two rainy seasons with a minor one from March to May, and a major one from September to November; two dry seasons, with the major one from December to February and the minor one from June to August. The landscape is characterized by the predominance of dense evergreen forest, with village lands displaying a variety of interlinked vegetation types: fields, fallow lands, cocoa and coffee plantations, isolated forest clumps, streams, and marshy hollows. The main villages of this HAT focus, Lambi and Bidjouka, lie on the right bank of the Mogue River. The farmers produce most of their own nutritional needs, plus a few market crops. Farming encampments are a key component of the farming system. Each family has its own water point. Most of the information regarding the human population was provided by a case-control study performed at the end of the twentieth century [10].

### The HATSim model

The ODD protocol is organized around three axes: Overview, Design concepts, and Details (ODD). These axes are divided into subparts that must be described precisely: purpose, state variables and scales, process overview and scheduling, design concepts, initialization, input, and submodels.

Distribution and behavior of human, tsetse, and animal (both wild and domestic) agents in this model followed what was observed in the field during several previous studies concerning tsetse fly and wild mammal populations [10, 11, 15, 19, 20, 24]. The observed densities of tsetse flies were all recorded taking into account each biotope (village, fields, water points, cocoa plantations, footpaths, deep forest, and shoals) by the capture point and their seasonal variations. All agents are present in spatial units that are characterized by a main environmental aspect, as described before. Finally, flies are the only agents we created, made to live, and die.

We tried to evaluate the epidemiological level of each epidemiological occurrence by defining a scale.

#### Purpose

We created HATSim to develop a model that can integrate the spatial complexity of a HAT focus in the simulations, such as the agents’ movements, as well as vectors or hosts, humans or animals, and the seasonal variations that directly impact the density of tsetse flies. The main purpose of HATSim is to predict the different scenarios of extension of the disease: in natural conditions without any public health action, with mass screening and treatment of the cases, and with such a public health intervention completed with vector control.

#### State variables and scale

##### Spatial units

The simulation space is defined as a 30 × 30 square grid covering an area of 36 km^2^. Each square or cell is 200 m long, allowing refined spatial resolution that can take into account the small size of the cultivated land and forest trails. Two hundred meters long also corresponds to the attractive diameter of a trap for tsetse flies. Each cell is characterized by an environmental attribute, and the cells are distributed according to previous field observations [16] into village (4%), crop field (10.5%), cocoa plantations (22.5%), encampments (2%), forest (40%), shallows (5.5%), water (11%), and footpaths and tracks (4.5%). Each attribute is associated with a specific color on the map. The scenes are PROX or NON PROX, which means that PROX cells correspond to those reserved for domestic animal divagation. PROX scenes include the village scenes and a perimeter of 400 m (2 cells) around the village.

##### Environment

The Bipindi region is characterized by four seasons; this seasonal variation has a great influence on the local tsetse fly population. A 50% decrease in the fly population was programmed for dry seasons, whereas a 50% population increase was programmed for rainy seasons. Distribution of tsetse flies was initialized according to results observed with fly captures in the different biotopes in the area of Bipindi [11].

##### Humans

In our simulation, the population of Lambi was stabilized around 500 inhabitants. Ten percent of the population was considered as sedentary (babies, elderly, or sick people). The presence of humans in these units is random, except for fields or plantations that have their owners. The remaining active population travels to the field, cocoa plantation, water point, encampment, and forest using footpaths, and as we said, their distribution is stochastic. During the second period of sickness, we considered that the patient would stay at home or be transferred to a medical center far from active tsetse flies and cannot constitute a reservoir. If diagnosed by the National Control Program, the individual is considered to be recovered; if not diagnosed, the patient will no longer be in the human population at the end of the second stage.

##### Animals

Massussi et al. [19, 20] recorded 31 wild mammal species in Lambi and determined their densities, including six known reservoir hosts of *T. b. gambiense*. Referring to the book “Mammals of Africa” [17], 59 species were identified in the area, that were able to be introduced into the model, including 28 rodents, 3 Pholidota, 8 Carnivora, 3 Cetartiodactyla, 15 primates, and 2 Artiodactyla. In line with Massussi’s estimation of infected wild mammals, we included the six potential reservoir species representing 47% of the whole wild mammal population. This results in 296 animals acting as potential reservoirs among a population of 600 wild mammals and 30 domestic animals and small livestock (pigs, goats, and sheep) that were identified in the village [21, 25]. The animal population is closed to the model and there are no entrances or exits programmed. Attributes and status are indicated in Table 2. Concerning the animal agents, distribution is stochastic and the wild ones are excluded from villages; we have rather focused on the definition of their territories and movements.

##### Tsetse flies

*Glossina palpalis palpalis* is the main sleeping sickness vector in the Bipindi area. For tsetse flies, the attributes are: sex, teneral state, age, pregnant female, hungry state, transmitting competence, and infected state (Table 2). When each fly is initialized, a counter for the duration of fasting, that will condition the bite for blood feeding, starts. In case of an infected first blood meal of a competent fly, a counter of incubation will start and run for 25 days before the fly is able to transmit the parasite. The methods are implemented in day steps, during which the flies are being born, biting, transmitting, growing, laying eggs, moving, and dying, and night steps (resting time). The vector population respects seasonal changes and the whole population is maintained in equilibrium.

The total tsetse population that an environment could support was estimated with *Glossina palpalis* in the forest area of the Ivory Coast [9]. By studying population dynamics, capture – recapture experiments have made it possible to determine a relationship between the density of flies caught in traps and the estimated total numbers of flies. This was all summarized by the equation *N* = *a* (Apparent Density per Trap per day)^*b*^, where *N* is the estimated population for one trapping site, and *a* and *b* are the constants defined by mark and recapture experiments. In our model, we kept *a* and *b* parameters obtained by Gouteux [*N* = 631.8 (1.8)^0.62^] with *G. p. palpalis* in villages of Ivory Coast [9], and used the apparent density per trap per day (ADT) observed in Lambi, as Müller et al. [23] did when carrying out the first ABM on HAT. The daily mortality rate (DMR) that could keep the vector population in equilibrium (about 3500 flies) was defined, after hundreds of simulations, as 0.0286 in this area.

We also had to determine the ambit of the flies. Considering the tropical forest environment of Lambi, which favors humidity and a great diversity of feeding hosts, and the study by Melachio et al. (2011) about the genetics of tsetse flies in Cameroon that identified panmictic subpopulations of *G. p. palpalis* [22], flies were programmed to have a range with a radius of 300 m in length, or a circle with a diameter of 3 cells in HATSim. The tsetse population is closed to the model and there are neither entrance nor exit movements.

##### Collectives

*The National Control Program or NCP* starts mass screening one week after T0 at TS84, followed by one screening every year, corresponding to five medical surveys in 5 years. To closely simulate the NCP interventions in the field, our simulations randomly covered 70% of the population.

*Vector control* was tested with a trapping device that covered all of the transmission scenes where humans can be found, except in the forest. This device implies the use of 200 traps. The programmed impact on the vector population follows what was observed in the field by Laveissière et al. in 1994 (e.g. a 90% decrease in the vector population in 2 months) [18].

#### Process overview and scheduling

The implementation of HATSim follows several steps: first, the initial instantiation (instance creation) allows defining of the space, the agents, their attributes, and the initial values that will be applied to each; following initial instantiation, the control step allows agents to operate in time according to a schedule, as well as interactions between agents (e.g. blood feeding and transmission). Time passes discretely with a time step corresponding to 2 hours, which is the estimated mean time spent on an activity by villagers; correspondingly, there are 21,900 time steps (TSs) in a 5-year period. The daily 24 hr are divided into day steps and night steps.

Tsetse flies are the only agents to really follow a schedule: we make them emerge, live (feeding, reproducing, and transmitting), and die (see Table 3). Daily, humans only return to sleep in the village after 6 time steps (TSs) spent moving randomly in the grid, except for field and plantation owners. Animals only move as programmed in Table 1, according to their size. However, both types of agents can be bitten by tsetse flies and be infected; in the same cell, there is a 20% probability for humans to be bitten, and 80% for animals. As soon as the parasite is transmitted to the host by a competent fly, a time counter is triggered to determine the slots in which the duration of the disease (two stages in humans) will be randomly defined.

**Table 1.** Attributes and status of human agents.

Attributes	Status
Infection level	Non-infected, 1st Period (randomly lasting from 1 to 4 years), 2nd Period (randomly lasting from 2 to 6 months).
Localization	Everywhere during the day, in the village by night Four hours a week by water points

**Table 2.** Attributes and status of animal agents.

Attributes	Status
Size	Small, medium or large
Nature	Domestic or wild
Territories	Radius of territories according to the size “PROX” cells only for domestic ones All cells except “VILLAGE” cells for wild ones
Movements	Depends on animal size Closed to the territory
Infection level	Infected or not infected
	Infection duration = 4 months max

**Table 3.** Attributes and status of tsetse fly agents.

Variables	Status
Sex	Male or female
Age	From 0 to 2 months (3 in the rainy season)
	Daily mortality rate (about 0.3%)
Infection state, teneral state, gravid female	Boolean
Parasite cycle	25 days from the infected blood meal to transmission ability, then transmission during each blood meal
Hunger level	Look for blood meal after 3 days of fasting Dies in 6 days without eating
Territory	Radius of 1.5 cell
Movements	One cell per TS, does not go into deep forest
Transmission competence	Randomly 2% of infected flies

Simulations were performed on a standard personal computer (PC) and data were recorded using MS Excel^®^. They were initiated by introducing 20 sleeping sickness cases at the first stage of the disease in the model (T0). The number of patients in this simulation corresponds to what was observed in 2000 by the Cameroonian NCP. Each parameter was tested using at least 100 simulations. For the analysis, the 21,900 TSs were reduced to weekly occurrences (every 84 TSs). Furthermore, the time steps corresponding to the NCP’s interventions were retained for epidemiological evaluations.

#### Design concepts

Taking into account the complexity of HAT transmission in the forest area of Bipindi, such as the distribution of the vector according to biotope and seasons, the mobility, in space and time, of the potential animal and human hosts, the variable duration of the reservoir, or the exceptional occurrences of competent vector for transmission, HATSim appears to be an appropriate tool to manage these constraints.

##### Emergence

Some human habits can be impacted by the disease: in HATSim, all the patients who enter the second stage of the sickness (neurological stage) will disappear from the system at the end of the second period (fatal cases or treatment in a hospital far from the village). We observed that in simulations without any public health screening and treatment, this leads to an average decrease of 14% of the human population.

##### Adaptation

Seasonal variations in the area imply significant changes in the DMR of flies, but this also means changes in the lifetime of tsetse flies: 3 months for those emerging in the rainy season against 2 months for those that emerge in the dry season. Looking for blood meals is also dependent on the hunger level: after one blood meal, a fly will not be hungry for 4 days; past this time, it will look for a new blood meal and will die 6 days after the last one if it cannot find any blood provider.

##### Interaction

**Figure 2.**
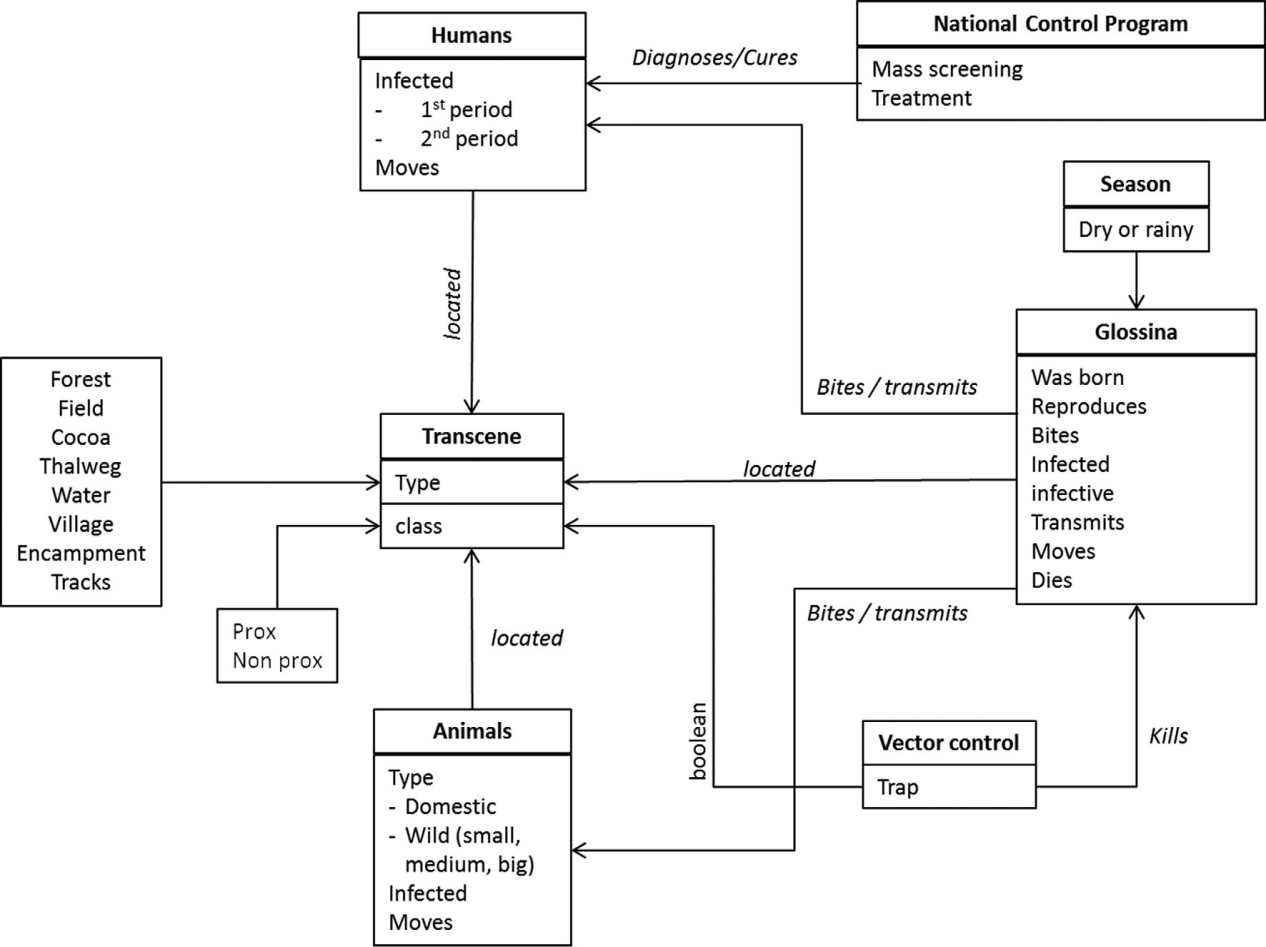
Organigram of interactions between agents in HATSim.

The only interactions between tsetse flies, humans, or animals are blood meals. For *glossinas*, this implies hunger, and for humans or animals it can be to become infected if they are bitten by a competent infected vector, or inversely it can be to infect a teneral fly by a first blood meal on an infected mammal. These interactions can take place in each cell (Fig. 2). Twenty percent of blood meals are taken on humans, all the others on animals.

##### Stochasticity

Stochasticity is applied to all active agents. *Glossinas* can be killed, due to the DMR that strikes the population randomly. In the same way, if blood feeding occurs mainly (80%) on animals, the choice of the blood donor is random; only 2% of infected vectors are considered to be competent for transmitting the parasite. Concerning humans, when the NCP performs screening of the human population, people are screened randomly according to the percentage defining the medical cover. Evolution of the disease in a patient is also determined in a stochastic way: the first stage can last from 1 to 4 years and the second stage from 2 to 6 months. Although territories are defined for most of the agents, the move from one cell to another at each TS is randomly applied.

##### Observation

During the simulations, the Cormas^®^ system allows the visualization of all agents’ movements on the spatial grid and of indicators at each time step (TS) to follow the dynamics of events such as: the number of patients in the first and second periods, the number of the human population (that can change if second-stage patients die), the number of tsetse flies, infected and infective flies, and the number of infected animals. These indicators allow us to calculate the prevalence of the sickness that is fundamental to evaluate epidemiological evolution.

#### Initialization

The values corresponding to the agent’s attributes can be modified before each simulation (Table 4).

**Table 4.** Units and initial values for each HATSim agent.

Agents	Attributes	Values	Sources
*Glossinas*	*n*	5000	Müller et al. (2004) [23]
	Daily mortality rate	0.2865	Defined by modeling by a fly population at equilibrium
	Sex ratio	1	Müller et al. (2004) [23]
Wild mammals	*n*	400	Massussi et al. (2009, 2010) [19, 20]
			Kingdon et al. (2013) [17]
	Size ratio (small/med./large)	0.65/0.25/0.1	Massussi et al. (2009, 2010) [19, 20]
			Kingdon et al. (2013) [17]
	Infection sensitivity ratio (small/med./large)	0.51/0.13/0.79	Massussi et al. (2009, 2010) [19, 20]
Domestic animals	*n*	30	Van Hoof et al. (1947) [28]
	Infection sensitivity ratio	100%	Penchenier et al. (2005) [25]
Humans	*n*	500	Census 1999
	No. of cases	20	NCP 2000
NCP	Medical cover rate	0.70	NCP 2000
Vector control	No. of traps	0–200	According to modeler

#### Submodels

This mainly concerns all the programming of the agents’ constraints. The DMR is the most sensitive parameter we had to test in order to obtain a fly population at equilibrium a few days after the simulations started. Tsetse flies are distributed on the grid according to the environmental attribute that was accorded to the cell; this was determined according to field studies. Humans have some constraints such as coming back to the village for night, going to a water-point 4 TSs a week, or going to their field or cocoa plantation if they are owners.

Movements, during a 5-year simulation, are limited to each agent’s territory as defined during the initialization. For each TS, they are limited to cell by cell for tsetse flies, medium and small animals. Flies cannot access an isolated “forest cell”, as observed in the field.

Times had to be under control for the infections process: beginning of the 25 day cycle of the trypanosomes in the fly, time of the sickness stages for humans, and the time of infection in animals; this needed specific programming.

#### Sensitivity tests

**Figure 3.**
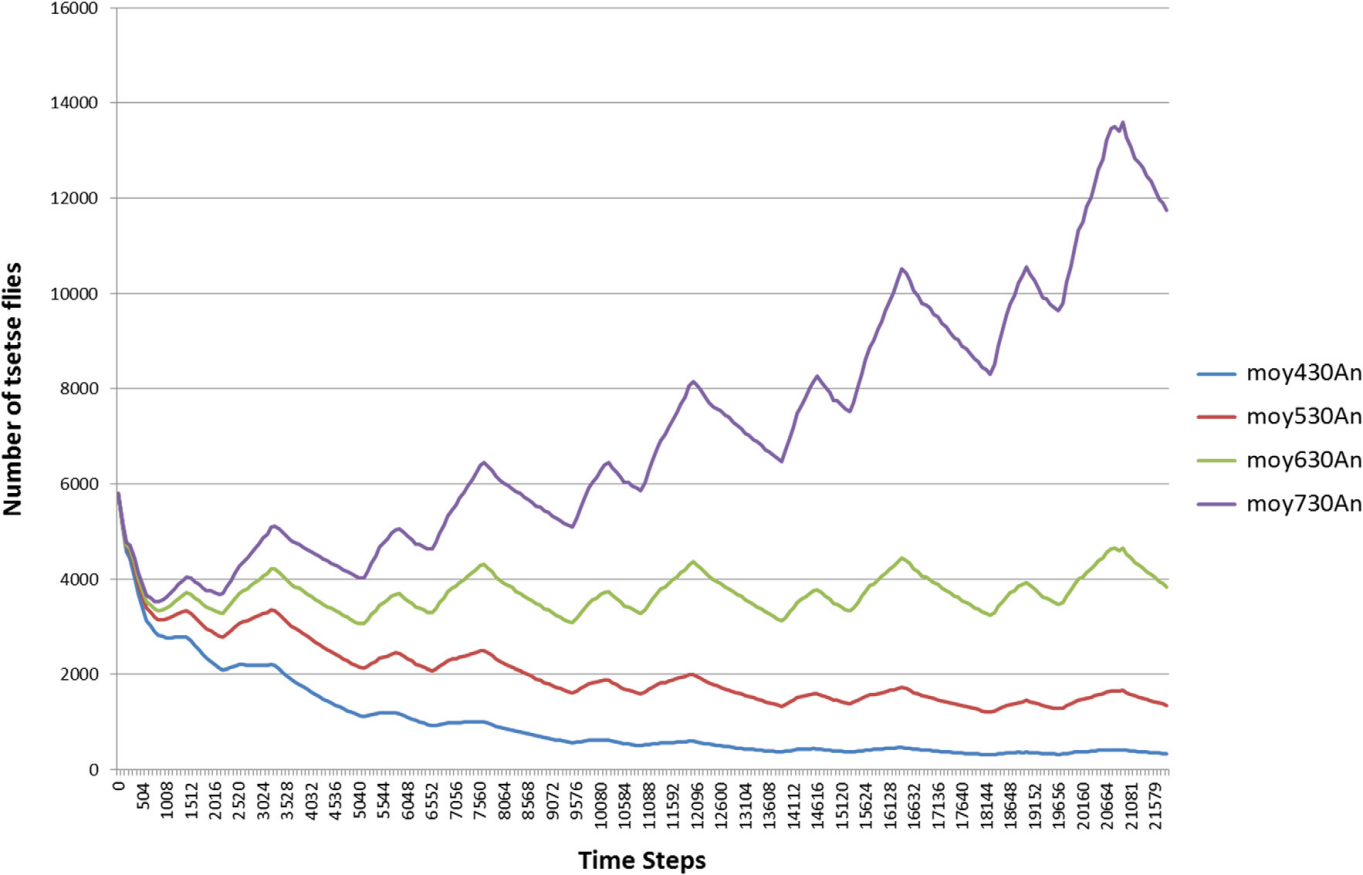
Sensitivity test on the average number of tsetse flies (Moy) in 20 simulations, according to different numbers (430, 530, 630 and 730) of animals (An).

One of the most important parameters is the equilibrium of the vector population, including the DMR, the seasonal variability, and the capacity of blood feeding, depending on the presence of humans or animals. Knowing the human numbers (500), that is almost constant, except during epidemic events, we had to estimate the number of wild mammals that could be able to maintain the equilibrium of the *glossinas*’ population (Fig. 3). Added to the 30 domestic animals, 630 mammals were the number we retained with a 0.0286 DMR.

**Figure 4.**
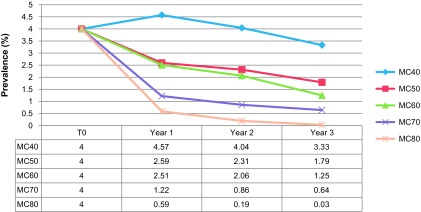
Sensitivity test about the evolution of the average prevalence of sleeping sickness during 3 years, using 5 different percentages (40, 50, 60, 70 and 80%) of medical covers (MC).

We tested the sensitivity of the model using different medical cover rates or and vector control devices. For the medical cover rates, we observed the evolution of prevalence of sleeping sickness with different medical cover rates (40%, 50%, 60%, 70%, and 80%), as exposed in Figure 4.

Medical cover was the only parameter that was modulated in these simulations. We initiate simulations reproducing the epidemiological situation observed in the field by the NCP in 2000, which diagnosed 20 HAT cases. Each medical cover was tested through 25 simulations over 3 years. We can observe that the prevalence curves in Figure 2 give coherent results in relation to the medical cover rates: the more people are tested, the more cases are found, and consequently the more the prevalence falls.

**Figure 5.**
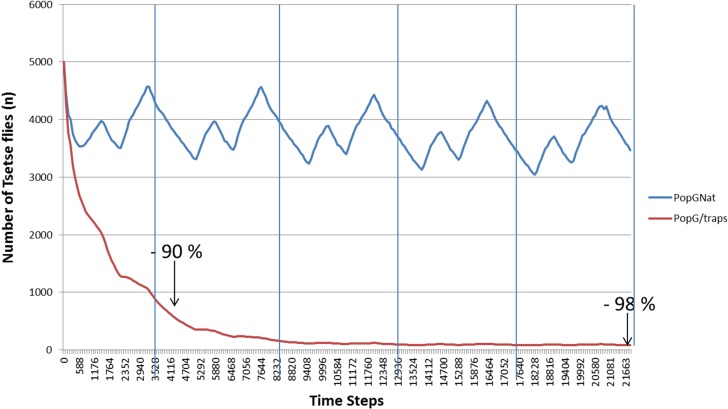
Comparison, over 5 years, of the evolution of the natural tsetse flies population (PopGNat) with a vector control device (PopG/traps).

We also tested the efficiency of the vector control device, using a density of 6 traps per km^2^ (Figure 5). When compared to the natural population of tsetse flies at equilibrium, we noticed that vector control works in the model and that we reached a 90% decrease of the fly population in one year and that could be maintained up to 5 years.

## Results

### Modeling the epidemiological evolution of HAT in Bipindi

The availability of epidemiological data in this area has allowed us to validate the predictive quality of our model, including the historical spread of the disease, the mass screening and treatment interventions of the National Control Program (NCP), and the numbers of humans in the first and second phases of the sickness, as well as the impact of vector control.

**Figure 6.**
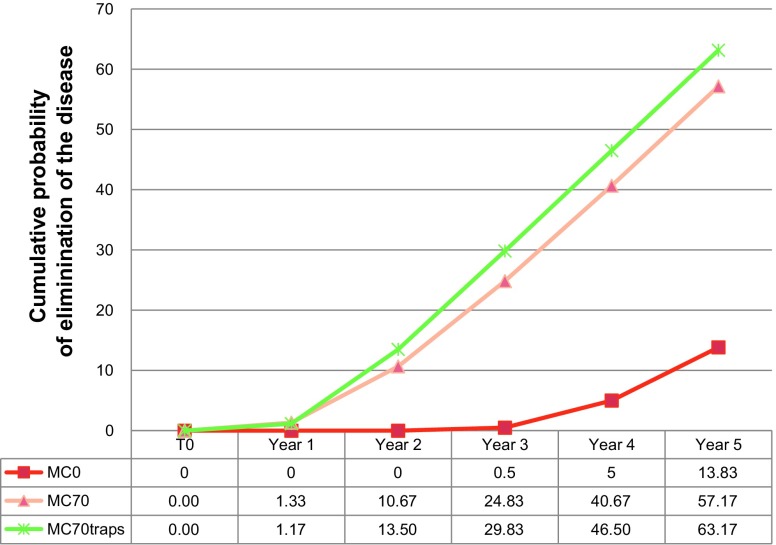
Cumulative probabilities of elimination of endemic events without any public health action (MC0), with a medical survey with 70% of medical cover (MC70), and with medical survey associated to vector control (MC70 traps).

When comparing the cumulative probabilities of extinction of the disease, with natural endemic evolution, with NCP interventions (70% medical cover), and with vector control (Fig. 6), we can see that vector control offers the highest probability of extinction events of the disease.

We first evaluated the natural expansion of the disease over a 5-year period in the absence of any public health intervention. This was performed in order to verify that the model was capable of proposing an endemic situation corresponding to the one actually observed by the first mass screening campaign carried out in the Bipindi focus (at the end of the twentieth century). We can conclude that without treatment of the cases, 14% of the population will disappear in 5 years.

We also evaluated the impact of the NCP in the beginning of the 2000s and recorded the endemic level that prevailed after each field survey. When comparing the occurrences of endemic events (R0 > 1) with and without NCP interventions during the 5-year period, it is apparent that the NCP interventions induce large decreases in the number and the level of endemic events during this period. This is seen as a relevant increase in elimination and, inversely, the drop of epidemic occurrences after the second medical campaign in year 2.

#### Impact of vector control

**Figure 7.**
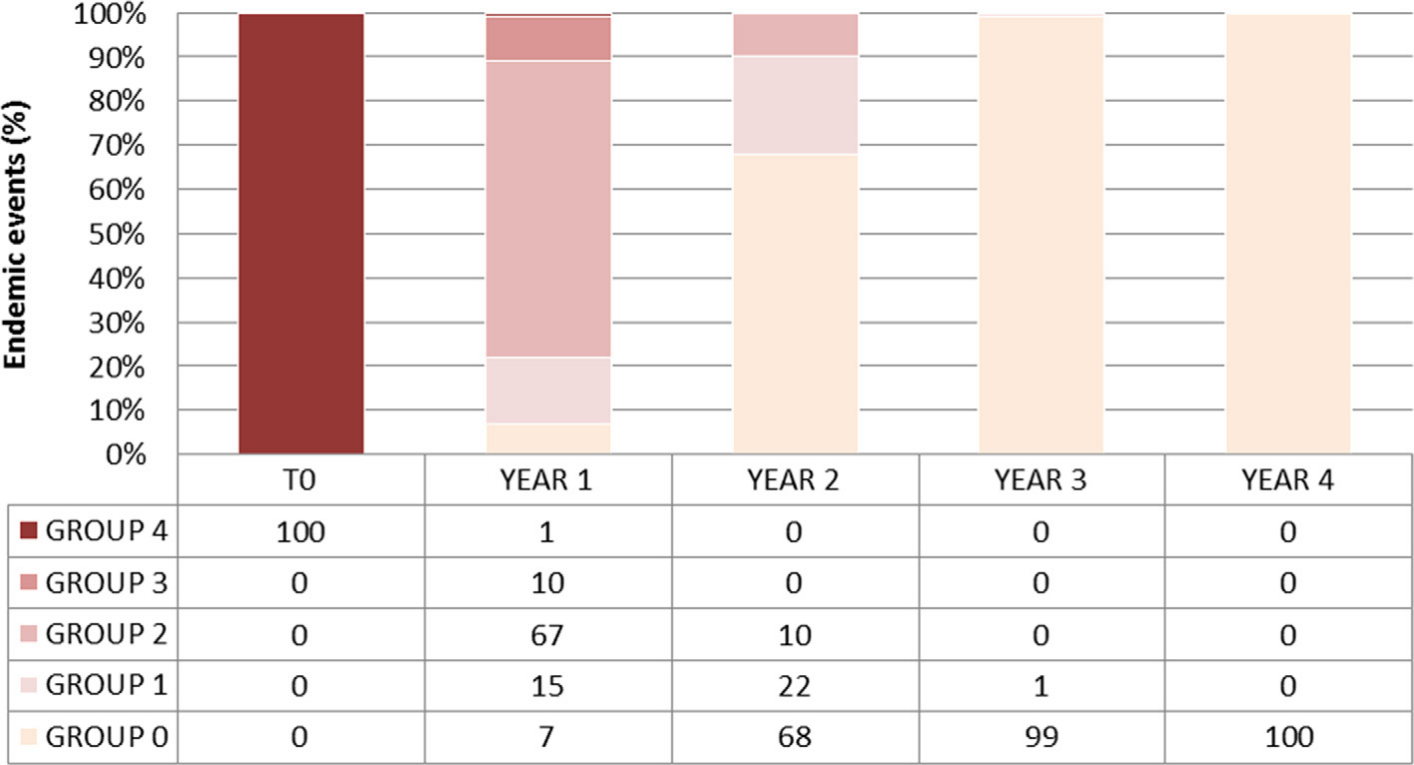
Occurrences of endemic events and of extinction of sleeping sickness in Lambi, with a 70% medical cover and vector control device (6 traps/km^2^). Group 0 = no case or elimination, group 1 = 1 case, group 2 = from 2 to 5 cases (low endemic level), group 3 = from 6 to 25 cases (high endemic level) and group 4 = more than 25 cases (epidemic situation).

Introducing both the NCP survey and trapping devices (6 traps per km^2^) in the model (Fig. 7) induces a significant difference (χ^2^ = 3.841, *p* value < 0.0001, DDL = 1) in the number of endemic events in the simulation without trapping. Furthermore, at year 3, sleeping sickness is eliminated in 99% of the events and there are no more endemic events (R0 > 1). Elimination is completed by year 4, when we cannot find even a single case.

## Discussion

Modeling HAT in Lambi provides greater detailed information on the natural endemic evolution of the disease in this area, the capacity of a public health program to eliminate the disease, and the effect of vector control on the disease. These results also demonstrate that this tool could easily be adapted to other sleeping sickness foci, once it has been developed on a wider scale.

Our simulation results are in agreement with those obtained by the NCP in Lambi between 2001 and 2011. Specifically, the first mass screening revealed 3.5% disease prevalence in Lambi in 2000; field observations as well as our simulations both indicated the disappearance of a serious endemic situation by the following year. In year 3, the disease was eliminated in 75% of the simulations and transmission only occurred in 14% of the events. In year 4, disease elimination occurred in 95% of the simulations and transmission occurred in only 3% of the events. Finally, in year 5, re-emergence occurred in only two events in groups 1 and 2. The absence of HAT cases in Lambi from 2004 to 2011 indicates that after 5 years of medical monitoring by the NCP, the disease was eliminated in Lambi, as also observed in our model in 98% of the simulations.

In contrast, the passive diagnosis of one case in the second stage of disease in 2012 from Lambi raises the question whether this is due to the maintenance of an animal reservoir or the arrival of a single case from another local village. The animal reservoir hypothesis was confirmed by Funk et al. [6], who found that HAT caused by *T. b. gambiense* could not be maintained in this focus without the contribution of an animal population. In the simulations, the animal reservoir is mainly limited by its duration (4 months), which is still sufficient for maintaining an endemic situation in a few simulations. However, we did not observe any autonomous cycle in animals, without any human cases, that was able to reinitiate an endemic situation. Therefore, we focused on a human reservoir instead. The epicenter of this HAT focus is principally composed of two villages, Lambi and Bidjouka, which present similar demographic, geographical, and social characteristics, and are regularly screened by the NCP. Nevertheless, additional cases were diagnosed between 2004 and 2012 in other villages. For example, five cases were identified in Ebimimbang, located 5 km south of Lambi, with which it shares a forest that was previously identified as a potential transmission [10].

Our results indicate that this ABM prototype for simulating the endemic evolution of sleeping sickness is efficient and offers a close approximation of reality, showing good predictive abilities. The results also demonstrate that the Cameroonian NCP reached elimination of the disease in the heart of the Bipindi focus, even though the maintenance of a reservoir in the neighboring villages favored the emergence of new HAT cases. Importantly, this shows that medical surveillance must pay particular attention to the perimeter of a focus. Improving the scale of the model will be the next step, and this will require incorporating new communities of agents linked to several villages into the simulations. HATSim is still in the early phases of development and must evolve to a larger scale, in order to integrate all the villages that could be included within a HAT focus. A better understanding of human population movements (inside and outside the area, and from one village to another) is a prerequisite for upgrading the scale of the model. Only then will the model be able to simultaneously integrate all villages within the focus into the simulations. Many other items relating to the model must still be clarified. For instance, the tsetse fly’s transmission competence, as well as its refractoriness to infection, remain unknown, as Davis et al. reported in 2011 [4]. We could add the estimation of the fly population or their average lifetime. The estimations of the wild mammal populations and of the animal reservoir constitute other speculative points; in particular, the uncertainty regarding the animal reservoir duration: although domestic animals have been the subject of several reports [21, 25, 28] very few studies have been conducted on wild animals. Consequently, we can only speculate on the duration of the reservoir of *T. b. gambiense* in wild fauna. This type of information can be sought in the field, but needs time and technical competence.

Although HAT elimination must still focus on the active reservoir before transmission spreads, our simulation shows that modeling the disease will also be an invaluable tool for predicting its elimination.

## References

[1] Alderton S, Noble J, Atkinson P. 2013 Simulating Sleeping Sickness: a two host agent-based model. Advances in Artificial Life, ECAL. Proceedings of the Twelfth European Conference on the Synthesis and Simulation of Living Systems, September 2–6, 2013, Taormina, Italy.

[2] Bousquet F, Barreteau O, Le Page C, Mullon C, Weber J. 1999 An environmental modeling approach: the use of multi-agent simulations, in Advances in environmental and ecological modeling. F Blasco, A Weill, Editors Elsevier: Paris p. 113–122.

[3] Bucheton B, MacLeod A, Jamonneau V. 2011 Human host determinants influencing the outcome of *Trypanosoma brucei gambiense* infections. Parasite Immunology, 33(8), 438–447. 21385185 10.1111/j.1365-3024.2011.01287.x PMC3427891

[4] Davis S, Aksoy S, Galvani A. 2011 A global sensitivity analysis for African sleeping sickness. Parasitology, 138(4), 516–526. 21078220 10.1017/S0031182010001496 PMC3282146

[5] Farikou O, Njiokou F, Simo G, Asonganyi T, Cuny G, Geiger A. 2010 Tsetse fly blood meal modification and trypanosome identification in two sleeping sickness foci in the forest of southern Cameroon. Acta Tropica, 116(1), 81–88. 20541513 10.1016/j.actatropica.2010.06.002

[6] Funk S, Nishiura H, Heesterbeek H, Edmunds WJ, Checchi F, Alderton S. 2013 Identifying transmission cycles at the human-animal interface: the role of animal reservoirs in maintaining gambiense human African trypanosomiasis. PLoS Computational Biology, 9(1), e1002855. 23341760 10.1371/journal.pcbi.1002855 PMC3547827

[7] Gouteux JP, Artzrouni M. 1996 Is vector control needed in the fight against sleeping sickness? A biomathematical approach. Bulletin de la Société de Pathologie Exotique, 89(4), 299–305. 9053054

[8] Gouteux JP, Artzrouni M. 2000 Persistence and resurgence of sleeping sickness caused by *Trypanosoma brucei gambiense* in historic foci. Biomathematical approach of an epidemiologic enigma. Comptes Rendus de l’Académie des Sciences III, 323(4), 351–364. 10.1016/s0764-4469(00)00145-1 10803346

[9] Gouteux JP, Buckland ST. 1984 Écologie des glossines en secteur pré-forestier de Côte d’ivoire : dynamique des populations. Cahiers O.R.S.T.O.M., série Entomologie médicale et Parasitologie, 22(1), 19–34.

[10] Grébaut P, Bodo JM, Assona A, Foumane Ngane V, Njiokou F, Ollivier G, Soula G, Laveissière C. 2001 Risk factors for human African trypanosomiasis in the Bipindi region of Cameroon. Médecine Tropicale, 61(4–5), 377–383. 11803830

[11] Grébaut P, Mbida Mbida JA, Kondjio CA, Njiokou F, Penchenier L, Laveissière C. 2004 Spatial and temporal patterns of Human African Trypanosomosis transmission risk in the Bipindi focus, in the forest zone of southern Cameroon. Vector Borne and Zoonotic Diseases, 4(3), 230–238. 15631068 10.1089/vbz.2004.4.230

[12] Grébaut P. 2012 Vector control in sleeping sickness foci today: has research been caught in its own trap? Journal of Medical Research and Science, 1(2), 48–56.

[13] Grimm V, Berger U, Bastiansen F, Eliassen S, Ginot V, Giske J, Goss-Custard J, Grand T, Simone Heinz SK, Huse G, Huth A, Jepsen JU, Jørgensen C, Mooij WM, Müller B, Pe’er G, Piou C, Railsback SF, Robbins AM, Robbins MM, Rossmanith E, Rüger N, Strand E, Souissi S, Stillman RA, Vabø R, Visser U, DeAngelis DL. 2006 A standard protocol for describing individual-based and agent-based models. Ecological Modelling, 198, 115–126.

[14] Hargrove JW, Ouifki R, Kajunguri D, Vale GA, Torr SJ. 2012 Modeling the control of trypanosomiasis using trypanocides or insecticide-treated livestock. PLoS Neglected Tropical Diseases, 6(5), e1615. 22616017 10.1371/journal.pntd.0001615 PMC3352824

[15] Herder S, Simo G, Nkinin S, Njiokou F. 2002 Identification of trypanosomes in wild animals from southern Cameroon using the polymerase chain reaction (PCR). Parasite, 9(4), 345–349. 12514949 10.1051/parasite/2002094345

[16] Jusot JF, de Vlas SJ, van Oortmarssen GJ, De Muynck A. 1995 Contribution of a mathematical model in the control of a parasitosis: the case of human African trypanosomiasis due to *Trypanosoma brucei gambiense*. Annales de la Société Belge de Médecine Tropicale, 75(4), 257–272. 8669973

[17] Kingdon J, Happold D, Butynski T, Hoffmann M, Happold M, Kalina J. 2013 Mammals of Africa, Volume 6, AC Black Publishers Ltd: London.

[18] Laveissière C, Grébaut P, Lemasson JJ, Meda H, Couret D, Doua F, Brou N, Cattand P. 1994 Les communautés rurales et la lutte contre la maladie du sommeil en forêt de Côte-d’Ivoire. WHO/TRY/94.1 (FRA).

[19] Massussi JA, Djieto-Lordon C, Njiokou F, Laveissière C, Van der Ploeg JD. 2009 Influence of habitat and seasonal variation on wild mammal diversity and distribution with special reference to the *Trypanosoma brucei gambiense* host-reservoir in Bipindi (Cameroon). Acta Tropica, 112(3), 308–315. 19732737 10.1016/j.actatropica.2009.08.027

[20] Massussi JA, Mbida Mbida JA, Djieto-Lordon C, Njiokou F, Laveissière C, Van der Ploeg JD. 2010 Diversity and spatial distribution of vectors and hosts of *T. brucei gambiense* in forest zones of Southern Cameroon: epidemiological implications. Acta Tropica, 114(1), 44–48. 20067756 10.1016/j.actatropica.2010.01.002

[21] Mehlitz D. 1986 Le réservoir animal de la maladie du sommeil *à Trypanosoma brucei gambiense*. Etudes et synthèses de l’I.E.M.V.T., Deutsche Gesellschaft für Technische Zusammenarbeit (GTZ): Eschborn, RFA, 18, 156.

[22] Melachio T, Simo G, Ravel S, De Meeûs T, Causse S, Solano P, Lutumba P, Asonganyi T, Njiokou F. 2011 Population genetics of *Glossina palpalis palpalis* from central African sleeping sickness foci. Parasite & Vectors, 4, 140. 10.1186/1756-3305-4-140 PMC3162924 21767402

[23] Müller G, Grébaut P, Gouteux JP. 2004 An agent-based model of sleeping sickness: simulation trials of a forest focus in southern Cameroon. Comptes Rendus Biologies, 327(1), 1–11. 15015750 10.1016/j.crvi.2003.12.002

[24] Njiokou F, Laveissière C, Simo G, Nkinin S, Grébaut P, Cuny G, Herder S. 2006 Wild fauna as a probable animal reservoir for *Trypanosoma brucei gambiense* in Cameroon. Infection, Genetics and Evolution, 6(2), 147–153. 10.1016/j.meegid.2005.04.003 16236560

[25] Penchenier L, Alhadji D, Bahébégué S, Simo G, Laveissière C, Cuny G. 2005 Spontaneous cure of domestic pigs experimentally infected by *Trypanosoma brucei gambiense*. Implications for the control of sleeping sickness. Veterinary Parasitology, 133(1), 7–11. 16076528 10.1016/j.vetpar.2005.04.034

[26] Rogers DJ. 1988 A general model for the African trypanosomiases. Parasitology, 97(Pt 1), 193–212. 3174235 10.1017/s0031182000066853

[27] Tchouomene-Labou J, Nana-Djeunga H, Simo G, Njitchouang GR, Cuny G, Asonganyi T, Njiokou F. 2013 Spatial and temporal variations relevant to tsetse control in the Bipindi focus of southern Cameroon. Parasites & Vectors, 6, 193. 23815985 10.1186/1756-3305-6-193 PMC3701558

[28] Van Hoof L, Henrard C, Peel E. 1937 Sur le rôle du porc indigène comme réservoir de *T. gambiense*. Comptes Rendus de la Société de Biologie, 129, 79.

[29] W.H.O. Control and surveillance of human African Trypanosomiasis. 2013 Report of a WHO Expert Committee. WHO Technical Report Series, 984, 237. 24552089

